# Outcome after renal transplantation utilizing octogenarian donors– retrospective single-center analysis within the Eurotransplant region

**DOI:** 10.1007/s00423-026-04255-4

**Published:** 2026-09-11

**Authors:** Dennis Kleine-Doepke, Oliver Beetz, Christian Hinze, Ulrich Zwirner, Cornelius van Beekum, Philipp Felgendreff, Markus Quante, Ulf Kulik, Moritz Schmelzle, Gerrit Grannas

**Affiliations:** 1 Department of General, Visceral and Transplant Surgery, Hannover Medical School, Carl-Neuberg-Str. 1, 30625 Hannover, Germany; 2 Department of General, Visceral, Pediatric and Transplant Surgery, Aachen University Hospital, Aachen, Germany; 3 Department of Nephrology and Hypertension, Hannover Medical School, Hannover, Germany

**Keywords:** Kidney transplantation, Octogenarian donors, Eurotransplant senior program, Graft function

## Abstract

**Background:**

A severe shortage of donors and organs remains the primary challenge in modern transplantation medicine. For kidney transplantation, prolonged time on the waiting list increases morbidity and mortality, particularly among elderly patients. Dedicated allocation initiatives, such as the Eurotransplant Senior Program (ESP), were established to shorten waiting times, and previous age limits for organ acceptance were raised to expand the donor pool for this specific patient population.

**Methods:**

This retrospective study evaluated all patients who received a kidney graft from an octogenarian donor between January 2006 and September 2023. Key outcomes assessed included patient survival, graft survival, and graft function.

**Results:**

A total of 71 kidney transplantations were included. Primary non-function occurred in five patients, and nine patients experienced delayed graft function. The 5-year recipient survival rate was 68.8%, and the 5-year death-censored graft survival rate was 65.3%. Graft function, assessed via median serum creatinine levels, remained stable throughout the follow-up period, although median creatinine values did not return to the normal range.

**Conclusions:**

Following meticulous donor selection, allocating kidney grafts from donors aged 80 years or older represents a viable strategy to expand the donor pool for ESP patients.

## Background

The scarcity of donor organs remains a critical challenge in modern transplantation medicine across all types of solid organs. In the clinical context of kidney transplantation (KTx), the gap between patients with end-stage renal disease (ESRD) on the waiting list and available donor grafts is steadily widening, resulting in prolonged waiting times. Recent reports indicate a median waiting time for KTx of 4.05 years in the U.S [1], 1.4 years in the U.K [2], 1.9 years in France [3] and 8.9 years in Germany [4]. Consequently, prolonged periods of dialysis increase morbidity, economic burden, and mortality in affected patients, particularly the elderly [5–8]. Due to an impaired baseline health status, multiple comorbidities, or death while on the waiting list, only 25% of kidney transplant candidates aged 65 to 74 years are transplanted within five years of initiating dialysis, compared to 58% and 73% in the age groups of 46 to 64 years and under 46 years, respectively [9, 10]. Furthermore, the cohort of elderly patients with ESRD has expanded significantly in recent years. Over the last two decades, ESRD prevalence in the elderly population has increased by 42.8% [11] and the global demographic changes projection by the United Nations anticipates a doubling of the proportion of individuals over 60 years between 2000 and 2050 (from 11% to 22%) [12].

In light of these circumstances, the Eurotransplant Senior Program (ESP, also known as the “old-for-old” program) was established in 1999 within the Eurotransplant region. The ESP allocates kidney grafts from donors > 65 years to recipients > 65 years to expand the potential donor pool. To minimize ischemic time for these expanded-criteria organs, allocation depends solely on the donor region and the recipient’s time on dialysis.

A rescue organ allocation is initiated if available donor organs cannot be allocated to any patient within five hours post-procurement, or if they are declined by more than five transplant centers. Furthermore, depending on graft quality or in the event of a rescue allocation from a donor > 75 years, transplant centers have the option of performing dual kidney transplantation (DKTx) into a single recipient. Five-year multicenter analyses demonstrated non-inferior outcomes for patients transplanted within the ESP compared to kidney graft recipients aged 60 to 64 years allocated via the standard Eurotransplant Kidney Allocation System (ETKAS) [13].

ETKAS prioritizes zero-HLA-mismatched kidneys regardless of donor and recipient location, allocating all other kidneys using a score based on HLA mismatches, waiting time, and expected cold ischemia time, while balancing donor and recipient numbers among participating countries.

Despite these efforts, waiting times for elderly patients remain substantial—at three to four years—in countries with a severe imbalance between waitlisted candidates and available donor grafts, such as Germany [13–15]. Consequently, evaluating strategies to further expand the donor pool, such as utilizing marginal or extremely extended-criteria organs for selected patients, is urgently needed. Here, we retrospectively evaluate the outcomes of selected patients at our center who underwent KTx with kidney grafts from deceased octogenarian donors (aged 80 to 89 years).

## Methods

This study presents a retrospective, single-center analysis of clinical outcomes in patients who underwent kidney transplantation (KTx) using grafts from deceased octogenarian donors.

### Recipients

All patients who underwent KTx at Hannover Medical School between January 2006 and September 2023 and received a deceased donor kidney graft from a donor aged 80 years or older were screened for inclusion. Recipients who underwent dual kidney transplantation (DKTx) using both organs from a single donor were excluded from the analysis. Relevant outcome parameters, as well as donor and recipient baseline characteristics, were retrospectively extracted from the electronic medical records.

### Definition of variables

Patient survival and death-censored graft survival were defined as the primary outcomes. Secondary outcomes included allograft function, assessed via serum creatinine levels at specific post-transplantation time points (1, 3, and 6 months, and 1, 2, 3, 4, and 5 years).

The analyzed donor variables comprised age, a history of hypertension or diabetes mellitus, body mass index (BMI), pre-procurement noradrenaline therapy, and terminal serum creatinine levels prior to organ retrieval. Recipient baseline variables included age, BMI, and time on dialysis. Additionally, human leukocyte antigen (HLA) mismatches, the maintenance immunosuppressive regimen, cold ischemia time (CIT), operative duration, and the incidence of biopsy-proven or clinically suspected acute rejection episodes were included in the analysis.

### Graft acceptance

Following the allocation of a deceased donor graft by Eurotransplant, the organ was accepted or declined through a joint consensus between an attending transplant nephrologist and an experienced transplant surgeon. The final decision regarding graft transplantability was made intraoperatively by the surgeon following back-table preparation and macroscopic evaluation of the organ. The primary institutional acceptance criteria for grafts from octogenarian donors included adequate baseline renal function, defined as an estimated glomerular filtration rate (eGFR) of more than 70 mL/min/1.73 m², and the absence of acute kidney injury or pre-existing chronic kidney disease.

### Surgical procedure and immunosuppression

All patients underwent standard heterotopic single-kidney transplantation in the iliac fossa. Standard maintenance immunosuppression consisted of a triple regimen comprising prednisone, tacrolimus, and mycophenolate mofetil. For induction therapy, basiliximab was administered to all but two patients; these two individuals received anti-thymocyte globulin (ATG) due to high pre-transplant sensitization, defined as a panel-reactive antibody (PRA) level of > 60%. Neither hypothermic nor normothermic machine perfusion was utilized in this cohort; all grafts underwent standard static cold storage.Neither cold nor warm machine perfusion of grafts was used in this cohort.

### Postoperative follow-up

Following hospital discharge, regular post-transplant monitoring was performed at our institutional outpatient transplant clinic. During the early postoperative phase, follow-up visits occurred biweekly and were gradually scaled back in frequency over time based on clinical stability.

### Statistics

Categorical variables were analyzed using the chi-square test, whereas continuous and ordinal data were evaluated using the Mann-Whitney U test. Statistical significance was defined as a p-value < 0.05. Statistical analyses and figure generation were performed using SAS JMP^®^ Pro (version 16.1.0; SAS Institute Inc., Cary, NC, USA).

## Results

Between January 2006 and June 2023, a total of 2,619 KTx procedures were performed at Hannover Medical School. Of these, 71 (2.7%) kidney grafts from 48 deceased octogenarian donors were allocated and included in this analysis.

The median donor age was 82 years (range: 80–88 years), and the median recipient age was 68.4 years (range: 60–79 years). On average, donors were 13.4 years older than their respective recipients (age difference range: 3.8–25.9 years). Regarding sex distribution, 43.7% of donors and 54.9% of recipients were male. Sex-matched transplantations were performed in 57.8% of cases, while 15.5% involved male-to-female and 26.8% female-to-male allocations. The median donor-to-recipient BMI ratio was 1.0 (range: 0.6–1.6), with a median donor BMI of 25.9 kg/m² (range: 19.5–34.9 kg/m²). The median donor serum creatinine level was 70.7 µmol/L (range: 33.6–141.4 µmol/L). Comorbidities among donors included hypertension in 69.0% and diabetes mellitus in 22.2%. Prior to organ procurement, the median noradrenaline dose administered to donors was 0.08 µg/kg/min (range: 0.0–1.3 µg/kg/min).

Among recipients, the median time on dialysis was 4.71 years (range: 0.87–9.65 years); one patient underwent preemptive transplantation. The median time on the waiting list prior to transplantation was 2.08 years (range: 0.02–8.98 years). In comparison, the median time between achieving active transplantable status (“t”) and actual transplantation—which can diverge from the total waiting time due to pending clinical examinations—was 0.52 years (range: 0.01–5.75 years).

The median cold ischemia time (CIT) of the donor kidneys was 705 min (range: 349–1,437 min), and the median operative duration was 116 min (range: 63–249 min). The median intensive care unit (ICU) stay was 2 days (range: 0–83 days), and the median hospital stay was 16 days (range: 7–149 days). The wide variance in ICU and hospital stays was primarily driven by two patients who experienced severe postoperative complications. One patient developed a mechanical ileus following simultaneous transabdominal resection of polycystic kidneys during KTx, requiring multiple surgical revisions for subsequent peritonitis. The second recipient suffered an acute myocardial infarction two days post-transplantation, leading to subsequent graft loss.

In two patients, maintenance immunosuppression was switched to belatacept due to poor graft function and biopsy-proven calcineurin inhibitor (CNI) toxicity. In nine patients, the CNI was converted to an mTOR inhibitor within the first 12 months post-transplantation.

**Fig. 1:**
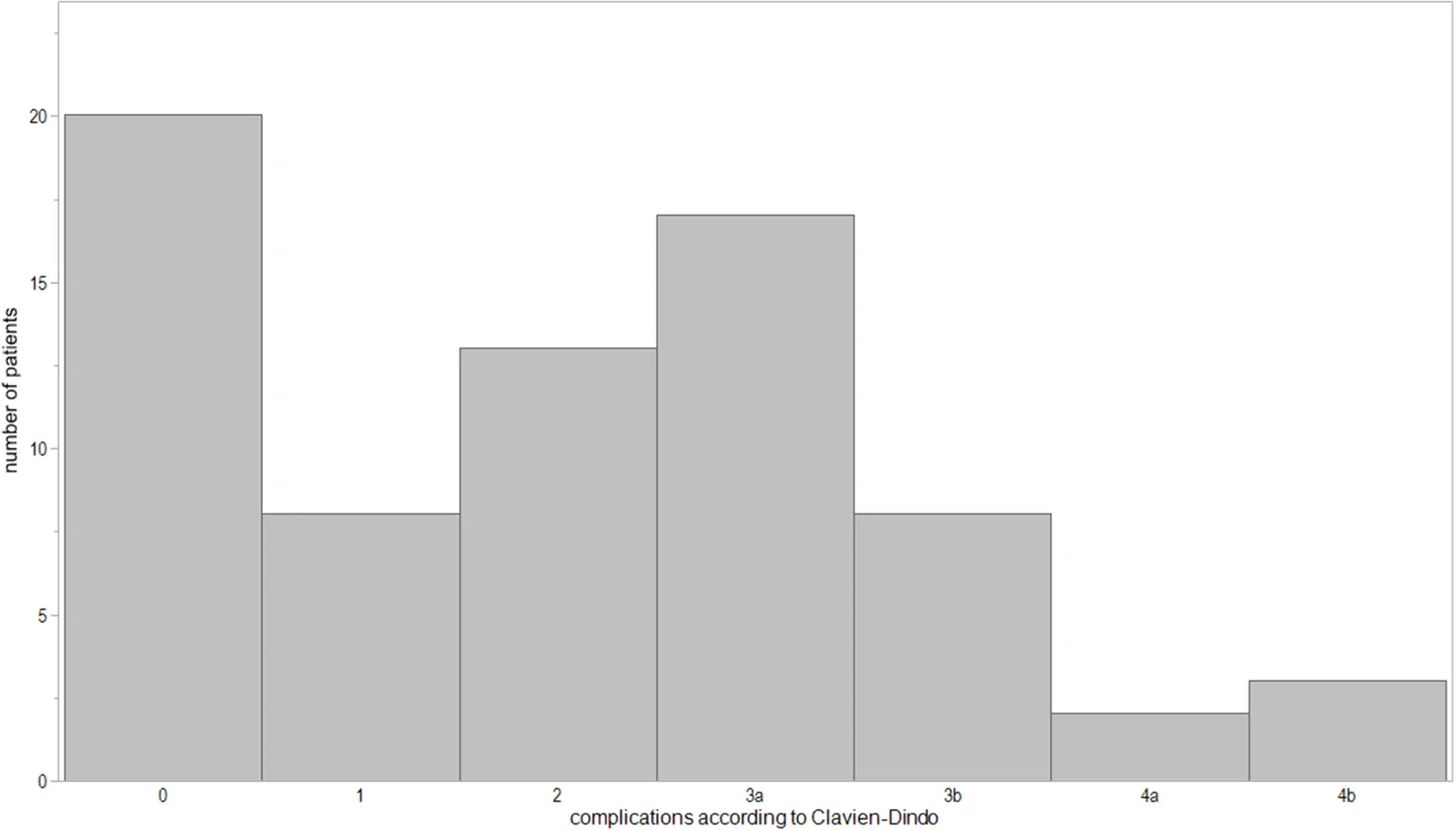
Shown are the number of complications according to the classification of Dindo et al

Postoperative complications classified according to Dindo et al. [16] are summarized in Fig. 1, tracking only the highest complication grade per patient. Primary non-function (PNF) occurred in five patients (7.0%; clinical details are provided in Table 1). Nine patients (12.7%) experienced delayed graft function (DGF) necessitating initial dialysis post-transplantation. Acute rejection episodes requiring treatment occurred in 13 patients (18.3%). While there was no difference in ICU stay between patients with or without DGF, the mean hospital stay was substantially longer in the DGF group (40.2 vs. 19.1 days), though this did not reach statistical significance.

**Table 1:** Characteristics of grafts with PNF (PNF: initial nonfunction, BMI: Body mass index, CIT: cold ischaemic time, SAB: subarachnoidal bleeding, ICB: intracranial bleeding, KTx: Kidney transplantation)

	Donor age years	Donor BMI Kg/m^2^	Donor diabetes mellitus	Donor cause of death	CIT minutes	Reason for PNF (if known)	Function of contralateral kidney/graft
#1	82	29	No	SAB	894	Myocardial infarction shortly after KTx	good
#2	80	29	No	ICB	546	Rejection with failure to treatment	not transplanted
#3	86	24	Yes	ICB	705	High catecholamine dose in donor, poor perfusion of graft	not transplanted
#4	82	23	No	ICB	580	Poor quality of perfusion during organ retrieval	PNF
#5	82	23	No	ICB	691	Poor quality of perfusion during organ retrieval donation	PNF

**Fig. 2:**
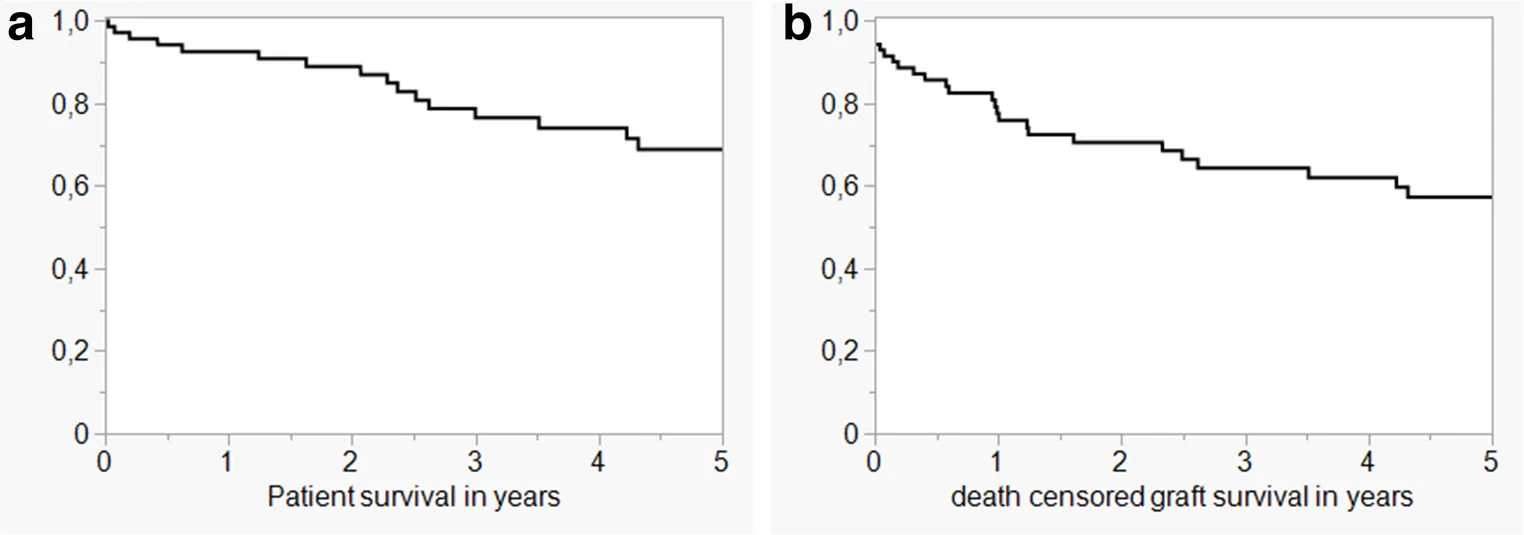
Five year patient (**a**) and death censored graft survival (**b**)

**Fig. 3:**
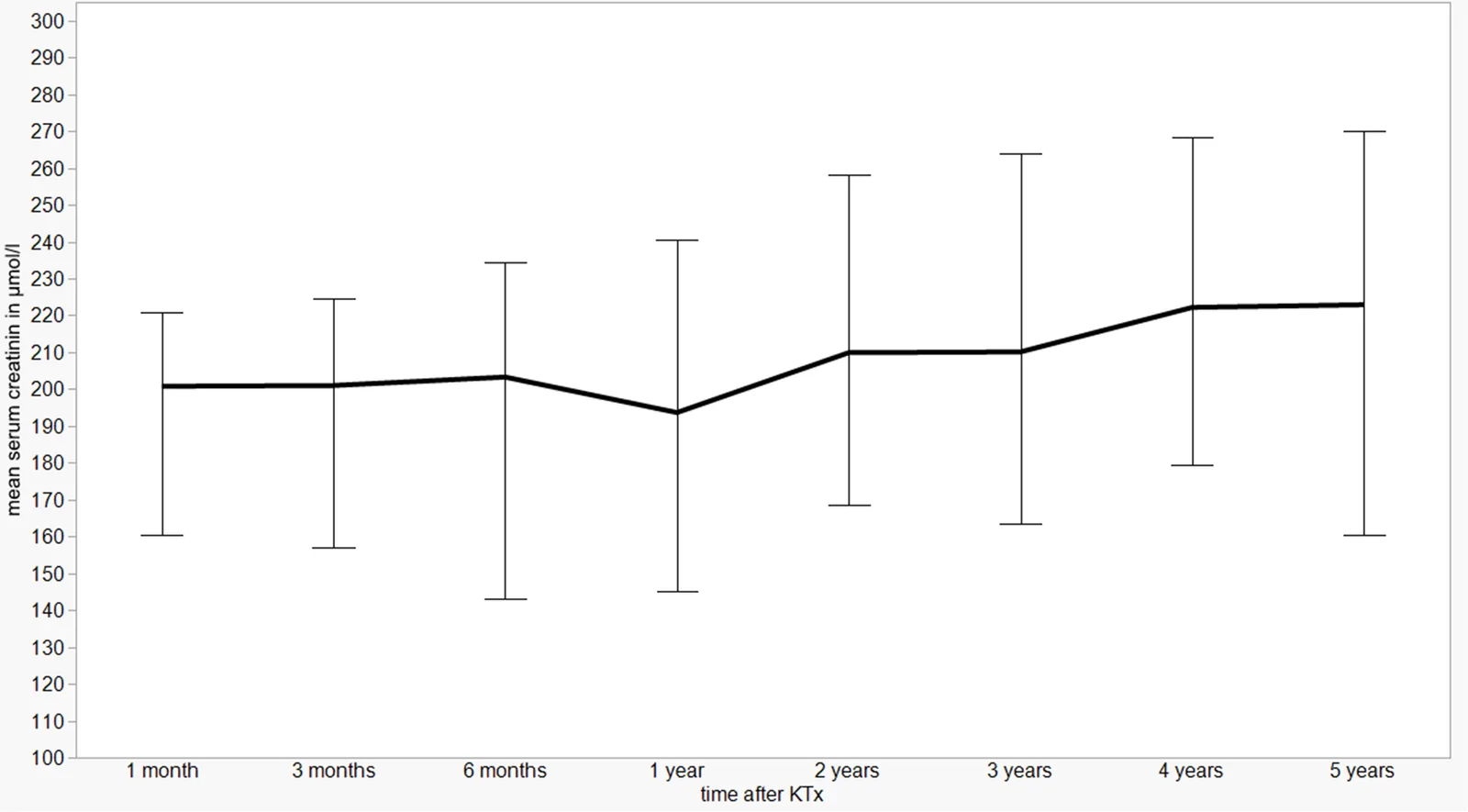
Mean serum creatinine values of functional grafts over times after kidney transplantation

The 5-year recipient survival rate was 68.8% (Fig. 2a), and the 5-year death-censored graft survival rate was 65.3% (Fig. 2b). Allograft function, assessed via median serum creatinine levels, remained stable throughout the follow-up period (Fig. 3), although median values did not return to the normal range. Notably, only two-thirds of the contralateral kidneys allocated to other transplant centers were successfully transplanted.

## Discussion

Kidney allocation in Germany is substantially influenced by regional factors [4]. The country is divided into 7 regions and 15 subregions; the present study was conducted at a transplant center within the “North” region and the “Hannover” subregion. A recent publication reported a median time of 9.7 years between dialysis onset and KTx in the “North” region, and 4.7 years for ESP patients within the “Hannover” subregion [4].

According to these data, utilizing deceased donor organs from octogenarians does not directly shorten the time on dialysis for the individual recipient, as we found no significant reduction in dialysis vintage for patients receiving an 80 + donor graft. However, expanding the donor pool by including ultra-elderly donors could reduce the overall waiting time for the entire cohort of ESP candidates by increasing the total availability of deceased donor organs.In a comparable German study, the primary non-function (PNF) rate was 10.8% [17], which aligns with our findings. Additionally, US data showed a 5-year graft survival rate of only 53.5% in a population aged 70 years or older receiving deceased donor KTx [18] further supporting our results.

Conversely, compared to another published German ESP cohort with exceptionally favorable outcomes [19], our 5-year death-censored graft survival rate was lower (65.3% vs. 93.5%). This discrepancy may be attributed to a higher rate of PNF in our cohort (7.1% vs. 1.6%). The survival benefit in the cited study likely stems from a highly selective graft and recipient matching process, with a substantially lower median donor age of 72 years.Compared to the ERA-EDTA registry data on kidney replacement therapy [20], both the 5-year recipient survival (68.8% vs. 86.5%) and graft survival rates (65.3% vs. 77.5%) in our study are inferior to general KTx survival outcomes. Nevertheless, our recipient survival rates remain markedly superior to the reported 5-year survival rate of 42.6% for patients remaining on maintenance dialysis [20]. Consistently, another study reported a 5-year survival rate of 48% for European patients with end-stage renal disease on dialysis [21]. Furthermore, 5-year survival rates on the KTx waiting list are even lower for patients aged > 70 years [18]. A separate study investigating 5-year outcomes in recipients aged 65 years and older reported a graft survival of 58% and an overall survival of 50% [22] both of which are inferior to the results achieved in our cohort. Finally, another German study demonstrated a 5-year patient survival rate of 61.2% among ESP patients [15] which is consistent with our findings (Fig. 2a). Consequently, patient survival following the transplantation of kidneys from octogenarian donors in this study is superior to the survival rates of patients remaining on dialysis reported in recent literature.While the 5-year recipient and graft survival rates after transplanting kidneys from donors aged 80 years or older may be lower than general ESP outcomes—which vary widely in recent literature—our data underscore that meticulous graft and recipient selection is essential to expand the transplantable organ pool while maintaining adequate outcomes. Retrospectively, donor selection appeared inadequate in three of the five PNF cases in our cohort (Table 1). In response, we have updated our institutional policy and no longer accept organs from donors requiring high-dose catecholamine therapy. Two additional kidneys from a single donor suffered PNF due to inadequate organ perfusion during procurement. This highlights that the technical quality of the procurement process must be rigorously verified.Furthermore, two patients required prolonged intensive care unit (ICU) stays due to concomitant diseases, and one patient experienced PNF following a perioperative myocardial infarction immediately after KTx, resulting in graft loss (Table 1). These cases underscore that the outcomes of ESP recipients are heavily influenced by numerous comorbidities beyond the surgical procedure itself. This emphasizes the critical importance of a thorough pre-transplant recipient evaluation in this vulnerable patient cohort.Another crucial outcome metric is health-related quality of life. A recent review demonstrated that elderly patients experience a more pronounced improvement in quality of life following KTx than those remaining on dialysis [23]. This further supports offering KTx to elderly ESRD patients using grafts from donors aged 80 years or older, particularly given the relatively stable long-term function observed in successful grafts (Fig. 3). A Dutch study indicated a lower probability of transplantation for candidates aged 64 years or older at the time of waitlisting [24]. While those authors advocate for expanding living donation in the elderly [24], utilizing deceased octogenarian donors represents an alternative strategy to secure KTx options for a patient group that otherwise faces low transplantation probabilities.Notably, Okidi et al. even advocate for living donation by octogenarian donors [25], which, however, does not reflect the current policy at our center.

Beyond clinical outcomes, the economic implications of kidney transplantation versus dialysis warrant consideration. An Italian study comparing pre- and post-transplant costs found that mean total costs per patient were €36,745.90 in the 12 months preceding KTx, compared to €22,622.40 in the first 12 months post-KTx, with the transplantation procedure itself costing €21,183.40 [26]. Based on these data, the surgical costs of KTx are amortized after two years of dialysis-free survival. Given the 5-year graft survival rate of over 60% in our study, KTx remains economically superior to dialysis, even when utilizing grafts from octogenarian donors.Fourteen patients in our cohort experienced either PNF or delayed graft function (DGF). This combined rate of 20% is lower than the 29.7% reported in general ESP literature [13]. While this reflects a clinical success, it stands in contrast to our reduced 5-year graft survival rates. Retrospectively, three of the five grafts that developed PNF (Table 1) would be rejected under our current criteria, which would conceptually minimize PNF incidence and improve long-term graft survival.

Our revised center policy is to accept kidney grafts from deceased donors over 80 years old only if they are free of major comorbidities—such as diabetes or coronary artery disease—and show no evidence of acute or chronic kidney injury.

Limitations of this study include its retrospective design. Comparing our results with current literature and registry data may introduce selection bias, even though published data define the clinical benchmarks. Additionally, a selection bias is inherent because only highly cleared patients are approved for waitlisting. Furthermore, organ acceptance relies not only on objective criteria but also on the subjective, real-time assessment of the retrieving surgeons and transplant nephrologists. To validate these findings and allow a more comprehensive evaluation, larger, multicenter studies are required.

## Conclusions

Although the transplantation of kidneys from octogenarian donors is associated with reduced graft survival compared to standard KTx cohorts, the outcomes in this study remain comparable to those of elderly recipients reported in the literature. Given that patient survival and quality of life are markedly superior to maintenance dialysis, utilizing deceased donors aged 80 years or older offers a justifiable strategy to expand the donor pool for ESP candidates. Through meticulous graft selection, these ultra-elderly organs can yield outcomes comparable to the broader ESP cohort, potentially reducing overall waiting list times. 

## Data Availability

The datasets used and/or analysed during the current study are available from the corresponding author on reasonable request.

## Abbreviations

BMI: body mass index
CIT: cold ischemic time
DGF: delayed graft function
ESP: Eurotransplant senior program
ESRD: end stage renal disease
ICU: intensive care unit
KTx: kidney transplantation
PNF: primary non-function
